# Retrospective Study of Aging and Sex-Specific Risk Factors of COVID-19 with Hypertension in China

**DOI:** 10.1155/2022/5978314

**Published:** 2022-06-28

**Authors:** Juan Wang, Yili Zhang, Kexin Li, KangJia Du, Xinyi Huang, Zifeng Zhou, Yan Ma, Shuzhen Guo, Yong Hou, Quntang Li, Hongming Xu, Jin Huang, Qiuhua Huang, Hui Na, Jingwei Wang, Xiaoyan Wang, Yanhua Xiao, Junteng Zhu, Hong Chen, Zhang Liu, Mingxuan Wang, Linsong Zhang, Wei Wang, Haitong Wan

**Affiliations:** ^1^School of Life and Health Sciences, Zhejiang Chinese Medical University, Hangzhou, China; ^2^School of Traditional Chinese Medicine, Beijing University of Chinese Medicine, Beijing, China; ^3^Dongzhimen Hospital, Beijing University of Chinese Medicine, Beijing, China; ^4^Institute of Basic Research in Clinical Medicine, China Academy of Chinese Medical Sciences, Beijing, China; ^5^The First Affiliated Hospital of Anhui University of Traditional Chinese Medicine, Hefei, Anhui, China; ^6^Chongqing Traditional Chinese Medicine Hospital, Chongqing, China; ^7^Department of Infectious Disease, Daqing Second Hospital, Daqing, Heilongjiang, China; ^8^Department of Traditional Chinese Medicine, The People's Hospital of Guangxi Zhuang Autonomous Region, Nanning, Guangxi, China; ^9^Department of Infectious Disease, Harbin Infectious Disease Hospital, Harbin, Heilongjiang, China; ^10^Department of Infectious Disease, Jinzhong Infectious Disease Hospital, Jinzhong, Shanxi, China; ^11^Department of Traditional Chinese Medicine, Mudanjiang Kangan Hospital, Mudanjiang, Heilongjiang, China; ^12^Department of Rehabilitation Medicine, The Affiliated Hospital of Putian College, Putian, Fujian, China; ^13^President's Office, The First Hospital of Qiqihar, Qiqihar, Heilongjiang, China; ^14^Department of Traditional Chinese Medicine, The First Hospital of Suihua City, Suihua, Heilongjiang, China; ^15^Department of Traditional Chinese Medicine, Suining Central Hospital, Suining, Sichuan, China; ^16^Department of Traditional Chinese Medicine, Hospital (T·C·M) Affiliated to Southwest Medical University, Luzhou, Sichuan, China; ^17^President's Office, Beijing University of Chinese Medicine, Beijing, China

## Abstract

**Background:**

Coronavirus disease 2019 (COVID-19) has been a global threat that pushes healthcare to its limits. Hypertension is one of the most common risk factors for cardiovascular complications in COVID-19 and is strongly associated with disease severity and mortality. To date, clinical mechanisms by which hypertension leads to increased risk in COVID-19 are still unclear. Furthermore, additional factors might increase these risks, such as the consideration of age and sex, which are of interest when in search of personalized treatments for hypertensive COVID-19 patients.

**Methods:**

We conducted a retrospective cohort study of 543 COVID-19 patients in seven provinces of China to examine the epidemiological and clinical characteristics of COVID-19 in this population and to determine risk factors of hypertensive COVID-19 patients. We also used univariable and multivariable logistic regression methods to explore the risk factors associated with hypertensive COVID-19 patients in different age and sex subgroups.

**Results:**

Among the enrolled COVID-19 patients, the median age was 47 years (interquartile range (IQR) 34.0–57.0), and 99 patients (18.23%) were over 60 years old. With regard to comorbidities, 91 patients (16.75%) were diagnosed with hypertension, followed by diabetes, coronary disease, and cerebrovascular disease. Of the hypertensive COVID-19 patients, 51 (56.04%) were male. Multivariable analysis showed that old age, comorbid diabetes or coronary heart disease on admission, increased D-dimer, increased glucose, and decreased lymphocyte count were independent risk factors associated with hypertensive COVID-19 patients. Elevated total bilirubin (odds ratio [OR]: 1.014, 95% confidence interval [CI]: 0.23–1.05; *p* = 0.043) and triglycerides (OR: 1.173, 95% CI: 0.049–1.617; *p* = 0.007) were found to be associated with elderly hypertensive COVID-19 patients. In addition, we found that decreased lymphocytes, basophil, high-density lipoprotein, and increased fibrinogen and creatinine were related to a higher risk of disease severity in male patients. The most common abnormal clinical findings pertaining to female hypertensive COVID-19 patients were hemoglobin, total bile acid, total protein, and low-density lipoprotein.

**Conclusions:**

Factors associated with increased risk of hypertensive COVID-19 patients were identified. Results to the different age and sex subgroups in our study will allow for better possible personalized care and also provide new insights into specific risk stratification, disease management, and treatment strategies for COVID-19 patients with hypertension in the future.

## 1. Introduction

Since December 2019, coronavirus disease 2019 (COVID-19) has spread around the world, causing a pandemic that threatens public health, and has resulted in a near complete halt in economic and social activities [1, 2]. By 2 May 2022, SARS-CoV-2 has infected 511,479,320 people and killed over 6.23 million, as reported by the World Health Organization (WHO) [3].

Currently, clinical characteristics of COVID-19 are being continuously described [4], and some multivariate models have been developed to predict mortality associated with COVID-19, based mainly on variables such as BNP, hypersensitive troponin I, and creatine kinase isoenzyme [5]. Further focusing on the risk factors involved, it has been proposed that patients with underlying comorbidities such as hypertension, diabetes, and obesity have worst outcomes [6]. In a cohort study of 1,590 patients from 575 hospitals, pre-existing hypertension was independently associated with severe COVID-19 (hazard ratio (HR) 1.575, 95% confidence interval [CI]: 1.07–2.32) [7]. Another study showed that hypertensive patients are prone to the highest morbidity (10.5%) following COVID-19 infection [8]. However, there are controversies regarding the association between hypertension and COVID-19. Recent findings suggest the lack of clinical evidence indicating that hypertension is a contributor to critical outcomes in COVID-19 patients [9]. Moreover, a meta-regression suggested that hypertension is a weaker comorbidity in increasing COVID-19 severity after adjustments of other confounding factors [10]; hence, future researches are essential to clarify this multifaceted and complex puzzle. Furthermore, men were reported to be affected by COVID-19 more easily than women in general [11]. Thus, there is a need to understand specific risk factors and representative characteristics of COVID-19 patients with hypertension, using well-designed clinical trials with large sample size.

As we all know, early prediction of disease course is important for the management of COVID-19. Risk stratification can help to accurately allocate medical resources and inform medical decision-making. However, there has been relatively rare study focused on the risk factors for specific management of COVID-19 combined with hypertension. Under the circumstance of the second wave of the pandemic, greater understanding of variation in COVID-19 risk in people with hypertension is still needed to tailor protection measures [12]. Thus, we believed that it gave us an opportunity to perform the retrospective longitudinal, multi-center study from a cohort of 543 confirmed COVID-19 cases in China. In the era of big data, a large volume of data can be obtained from electronic healthcare records (EHR), which causes the curse of dimensionality. In this study, we extracted variables from the EHR, including demographic features, clinical data, and clinical outcomes of COVID-19 patients with hypertension. We also compared the characteristics and risk factors of the hypertensive COVID-19 patients in different age and sex subgroups. We hope these findings will provide new insights into specific risk stratification, in-depth disease management, and personalized treatment strategies for COVID-19 patients with hypertension.

## 2. Methods

### 2.1. Participants

A total of 543 patients from 10 hospitals in China were included in this study in early January 2020. The diagnosis of COVID-19 was made according to the Guidelines of the World Health Organization (interim) and National Health Commission of the People's Republic of China (Trial Version 5) [13, 14] and was further confirmed by high-throughput sequencing or real-time reverse transcriptase polymerase chain reaction (RT-PCR) results of nasal and pharyngeal swab samples [15]. This study was approved by the National Administration of Traditional Chinese Medicine and the Provincial Administration of Traditional Chinese Medicine and Ethics Committee of the 10 participating hospitals. We collected first-hand data on this emerging infectious disease, and the requirement for the written informed consent was waived consequently.

### 2.2. Data Collection

Through tracing the disease process of COVID-19 and various comorbidities, this cohort study was aimed at recognizing risk factors in the process and providing clues to possible pathogenesis. Before recruitment, all investigators in our research team completed specific trainings to be familiar with the aims of the study. Clinical information including demographic data, symptoms and signs, laboratory findings, CT imaging data, treatment strategies, and clinical outcomes was collected from the enrolled COVID-19 patients on admission and during hospitalization. Routine blood examination, lipid levels, coagulation function, myocardial enzymes, liver and renal function, and immune function were analyzed accordingly. However, due to the pressing need to treat patients urgently, not all participants had complete data in this real-world study.

### 2.3. Definition

According to the Joint National Committee on Prevention, Detection, Evaluation, and Treatment of High Blood Pressure, hypertension was defined as sustained systolic blood pressure (SBP) ≥ 140 mm Hg and/or diastolic blood pressure (DBP) ≥ 90 mm Hg measured in clinic, or having a history of taking antihypertensive medications [16]. Type 2 diabetes mellitus (T2DM) was defined according to the Guidelines of the American Diabetes Association or diagnosed if the subject was on antidiabetic medications [17].

### 2.4. Statistical Analysis

All clinical data were expressed as medians and interquartile ranges (IQRs) based on variable types. For categorical variables, Chi-square test or Fisher's exact test was used to analyze frequency and percentage for comparison between the hypertensive and non-hypertensive groups. Student's *t*-test or Mann-Whitney *U* test was used to analyze continuous variables [18]. Further analysis of the collected data was carried out to evaluate the impact of aging (≥60) and different gender. To explore the risk factors related to hypertensive COVID-19 patients, univariable and multivariable logistic regression models were conducted. Baseline variables that were considered clinically relevant or showed a univariate relationship with outcome were analyzed using multivariable regression model [19]. Candidate variables with *p* values less than 0.1 on univariable analysis were included in the multivariable model [20]. Given the number of events available, variables were chosen carefully for inclusion to ensure a parsimonious model. Consequently, a few variables were excluded because the extent of missing data for these variables was too limited for the analysis. Risk factors of hypertensive COVID-19 patients of different age and sex were also analyzed using above methods. All statistical analysis was carried out with SPSS 23.0 software, and a *p* value < 0.05 was considered to be statistically significant.

## 3. Results

A total of 543 patients had a positive test result for SARS-CoV-2 within the study period.  Table 1 presents the main demographic and clinical characteristics of hospitalized COVID-19 patients in both hypertensive and non-hypertensive groups. Of these patients, the median age was 47 years (IQR 34.0–57.0), and 99 patients (18.23%) were over 60 years old. There was no significant difference in the sex ratio of both groups. Based on clinical assessment of severity, 53 patients were classified as severe COVID-19 cases according to the Guideline. Various comorbidities such as diabetes (8.47%), coronary heart disease (2.76%), and cerebrovascular disease (1.66%) were also analyzed. Cough (50.28%), fever (40.70%), and dry cough (31.86%) were the most common symptoms and signs. Majority of the patients had bilateral ground glass opacity in their chest CT scans, while a lesser percentage (18.05%) of the patients had unilateral ground glass opacity. Significant differences in background characteristics including lymphocyte count < 0.8 × 10^9^ per L, basophil percentage, hemoglobin, red blood cell distribution width SD, and urea/creatinine ratio between the two groups, as shown in Table 1. Medication details and other laboratory findings are shown in supplementary Table 1.

In addition, the characteristics of hypertensive COVID-19 patients were depicted according to age and sex groups. Triglycerides, total bilirubin, indirect bilirubin, D-dimer, red blood cell distribution width SD, and red blood cell distribution width CV in elderly patients were higher than those in non-elderly patients, while calcium level was lower in elderly patients (Table 2). With regard to sex, male patients had much higher hematocrit, total protein, globulin, glucose, and fibrinogen levels and lower total bile acid level, as compared with female patients. The occurrence of fever was higher in male hypertensive COVID-19 patients (52.94%) than in female counterparts (30.00%) (Table 3).

Table 4 shows the related risk factors of hypertensive COVID-19 patients. Old age, comorbid diabetes or coronary heart disease, D-dimer, glucose, lymphocyte count, red blood cell distribution width SD, and thrombin time were significantly associated with the hypertension status of the study subjects (*p* < 0.05). In the multivariable logistic regression analysis, we found that total bilirubin and triglycerides were associated with elderly hypertensive COVID-19 patients (Table 5).

Risk factors for male and female hypertensive COVID-19 patients are described as follows. Decreased lymphocytes, basophil, and high-density lipoprotein and increased fibrinogen and creatinine were found to be related to a higher risk of disease severity in male patients (Table 6). The most common abnormal clinical findings pertaining to female hypertensive COVID-19 patients were hemoglobin, total bile acid, total protein, and low-density lipoprotein (Table 7).

## 4. Discussion

With an exponential growth of COVID-19 cases worldwide, it is generally believed that hypertension could easily lead to certain types of infection and death [21]. Previous studies have identified that multiple complications were associated with a general increase in mortality and morbidity of COVID-19 [22, 23], and the most prevalent comorbidities were hypertension and diabetes. In this retrospective study conducted in 10 hospitals in China, hypertensive individuals accounted for 16.75% of all enrolled patients. They were more vulnerable to develop other complications, such as diabetes, coronary disease, and cerebrovascular disease, probably because hypertensive patients were usually older and tend to have more comorbidities. Pre-existing cardiovascular diseases including myocarditis, acute cardiac damage, and arrhythmias have also been reported to be associated with hypertensive COVID-19 patients. In this context, clinical studies are needed to identify high-risk individuals with hypertension and to develop potential therapies while exploring the underlying clinical mechanisms.

When it comes to pathogenicity in hypertensive patients, functional imbalance of the RAS and endothelial dysfunction should be given more attention [12]. In addition, hypertension was reported to be associated with a proinflammatory state, which includes higher levels of chemokines and cytokines [24–26]. In this study, we found that laboratory indicators such as lymphocyte, neutrophil, hemoglobin, cystatin C, urea/creatinine, glucose, and low-density lipoprotein were markedly correlated with hypertensive patients. These characteristics were hypothesized to be crucial in the infection and progression of COVID-19. The most typical features of COVID-19 include lymphopenia and neutropenia, which are related to strong inflammatory storm and poorer prognosis [27–29]. Patients with hypertension were more likely to have lymphopenia in our study. Therefore, as illustrated by our clinical results, we propose that the proinflammatory state favored by RAS imbalance might be the center of COVID-19 pathophysiological mechanisms. Elevated urea/creatinine ratio, reduced glomerular filtration rate, and lower cystatin C levels were manifested in hypertensive patients and might be indicative of cardiac, hepatic, and nephritic injures. Previous studies also showed that liver and kidney injuries were more prevalent in severe cases than in mild cases of COVID-19 [30].

This retrospective cohort study identified several risk factors for hypertensive COVID-19 patients on univariable and multivariable analysis. In particular, old age, comorbid diabetes or coronary heart diseases, lymphocyte count lower than 0.8 × 10^9^ per L, thrombin time, and D-dimer levels greater than 0.5 *μ*g/L on admission were associated with hypertensive COVID-19 patients. In the blood routine test of novel coronavirus pneumonia, the number of lymphocytes tends to decrease. If a large number of viruses accumulate in the lungs, lymphocytes, the main inflammatory cells responsible for clearing the human virus, will accumulate in the lungs, consequently reducing the number and proportion of lymphocytes in peripheral blood. When evaluating the severity of these patients, the number, absolute value, and proportion of lymphocytes are very important indicators of severe inflammatory reaction due to invasion by the virus [31]. Symptoms, age, and baseline comorbidities of patients are also very important factors for disease prognosis. Taken together, these findings illustrate a preliminary hypothesis of lipid dysregulation, hypercoagulative state, and hepatorenal damage that occurs simultaneously in hypertensive COVID-19 patients.

It has become increasingly clear that aging was an independent predictor of mortality in patients with COVID-19 [18, 32]. Age-dependent decrease in cellular and humoral immune response was previously reported in elderly patients, especially in terms of adaptive immunity [33]. In our study, the high risk of elderly hypertensive patients might be attributed to their poor overall health and an increase in the number of complications. Bilirubin, a supposed potent substance in scavenging hydrogen peroxide radicals, provides antioxidative effects. However, previous findings suggested that higher levels of total and indirect bilirubin in old patients were hazardous factors of hypertension [34]. Severe lipid disorder (including higher triglycerides, low-density lipoprotein, and total cholesterol), elevated D-dimer, electrolyte disorders, and inflammation were observed in old patients. Therefore, as the aging tendency of the COVID-19 patients accelerates, the elderlies are becoming an increasingly important subpopulation that requires special attention in light of health and social issues.

According to the previous study, hypertension is more common in men, and male is an independent cardiovascular risk factor [35, 36]. Despite the differences in risk factors, there is limited data regarding sex-specific screening strategies for COVID-19 patients with hypertension. Therefore, the indicators of liver and kidney functions, inflammation, and immune system in COVID-19 patients with hypertension were analyzed with respect to sex differences.

A higher proportion of male patients presented with fever symptoms in this study. The findings of this study discovered significantly lower amounts of bile acid excretion in male patients with COVID-19 and hypertension. Total bile acid (TBA) levels are used clinically as a sensitive and reliable indicator of hepatobiliary diseases, and lower TBA concentration (less than the median 3.6 *μ*mol/L) was independently and significantly associated with the presence and severity of coronary artery disease [37].

In addition, the blood glucose level of male patients was higher than that of female patients. Current studies indicate that upper-normal glucose levels may be an additional risk factor for hypertension [38]. Upper-normal glucose levels and mild hyperglycemia are frequently observed in patients with insulin resistance; old age, sedentary lifestyle, obesity, hypertension, and dyslipidemia are common risk factors for insulin resistance [38]. Elevated plasma fibrinogen levels in male patients are associated with essential hypertension and may contribute to the development of atherosclerotic disease in these patients [39]. In conclusion, men tend to have more traditional risk factors, whereas women often present with less traditional risk factors. On one hand, a female probably benefits from the protective effects of estrogen [40, 41], while on the other hand, ovarian hormones can reduce plasma renin levels and angiotensin-converting enzyme activity, thus leading to an anti-inflammatory profile in women [42]. All the results suggest that the health surveillance and preventions for male COVID-19 patients with hypertension should be strengthened to avoid severe inflammation as well as the deterioration of liver and kidney functions, while in female cases, more attention should be given to abnormalities within the blood system as well as electrolyte disorders. Although there are numerous similarities in risk factors between the sexes, there are sex-related differences in the underlying physiology that can affect personalized clinical decision-making in the treatment of hypertensive COVID-19 patients.

Despite the importance of the aforementioned findings, the current study, however, has some limitations. For example, clinical data in our study was collected under urgent circumstances, sometimes resulting in the incomplete collection of certain important indicators, which might lead to bias. Hence, one cannot exclude the possibility that hypertension was complicated with COVID-19. Furthermore, as a retrospective study, the relationship between observed factors and conclusions is exploratory, and its causality needs to be further confirmed by prospective study.

## 5. Conclusion

Hypertensive patients are more vulnerable to various complications of COVID-19. Age and sex are important facets to a hypertensive COVID-19 patient's healthcare experience. In-depth researches with an accurate representation of the general population are of increasing significance to providing the best possible personalized patient care. Large-scale studies that consider all potential biasness and confounding factors are warranted in the near future to affirm the link between pre-existing hypertension and COVID-19 severity, so as to better devise personalized treatment for COVID-19 patients with hypertension.

## Figures and Tables

**Table 1:** Demographic, clinical, laboratory, and radiographic findings of COVID-19 patients on admission.

	Total (*n* = 543)	Patients with hypertension (*n* = 91)	Patients without hypertension (*n* = 452)	*p* Value
*Demographics*				
Age, years	47.00 (34.00–57.00)	47.00 (30.00–57.00)	47.00 (35.00–57.00)	0.633
>60	99 (18.23%)	17 (18.68%)	82 (18.14%)	0.903
≤60	444 (81.77%)	74 (81.32%)	370 (81.86%)	
Sex				
Male	267 (49.17%)	40 (43.96%)	227 (50.22%)	0.276
Female	276 (50.83%)	51 (56.04%)	225 (49.78%)	
Disease severity				
Mild	67 (12.34%)	16 (17.58%)	51 (11.28%)	0.102
General	423 (77.90%)	68 (74.73%)	355 (78.54%)	
Severe	53 (9.76%)	7 (7.69%)	46 (10.18%)	
Comorbidity				
Diabetes	46 (8.47%)	18 (19.78%)	28 (6.19%)	<0.001
Coronary heart disease	15 (2.76%)	9 (9.89%)	6 (1.32%)	<0.001
Cerebral vascular disease	9 (1.66%)	4 (4.40%)	5 (1.11%)	0.025
Common Symptoms				
Cough	273 (50.28%)	53 (58.24%)	220 (48.67%)	0.096
Fever	221 (40.70%)	39 (42.86%)	182 (40.27%)	0.646
Dry cough	173 (31.86%)	30 (32.97%)	143 (31.64%)	0.804
*Laboratory findings*				
Lymphocyte count, ×10^9^/per L	1.38 (0.95-1.80)	1.37 (0.96-1.78)	1.50 (0.89-1.94)	0.175
<0.8	82 (15.10%)	17 (18.68%)	65 (14.38%)	0.048
0.8–4	398 (73.30%)	61 (67.03%)	337 (74.56%)	
>4	7 (1.29%)	3 (3.30%)	4 (0.88%)	
Neutrophil count, ×10^9^/per L	3.71 (2.78–5.31)	3.66 (2.69–5.22)	4.40 (3.00–6.19)	0.234
<1.8	35(6.45%)	3 (3.30%)	33 (7.30%)	0.032
1.8–6.3	366 (67.40%)	57 (62.64%)	309 (68.36%)	
>6.3	76 (14.00%)	18 (19.78%)	58 (12.83%)	
Basophil percentage, ×10^9^/per L	0.26 (0.10–0.40)	0.22(0.13–0.36)	0.25 (0.08–0.40)	0.037
<1	401 (73.85%)	73 (80.22%)	328 (72.57%)	0.005
≥1	5 (0.92%)	0 (0%)	5 (1.10%)	
Hemoglobin, g/L	134.00 (123.07–148.48)	132.03 (123.11–147.15)	135.78 (123.04–148.23)	0.014
Urea/Creatinine	61.63 (5.55–90.47)	69.89 (38.16–96.09)	44.14 (0.07–68.36)	0.024
Cystatin C, mg/L	0.94 (0.80–1.13)	1.00 (0.33–1.23)	0.93 (0.21–1.35)	0.240
<0.54	2 (0.37%)	0 (0%)	2 (0.44%)	0.012
0.54–1.5	181 (33.33%)	34 (37.36%)	147 (32.52%)	
>1.5	11 (2.03%)	0 (0%)	11 (2.43%)	
Glomerular filtration rate	111.25 (102.37–120.79)	110.31 (101.15–121.21)	111.62 (103.73–119.71)	0.759
<90	6 (1.10%)	0 (0%)	6 (1.33%)	0.013
≥90	63 (11.60%)	10 (10.99%)	53 (11.73%)	
Low-density lipoprotein, mmol/L	2.40 (1.96–2.94)	2.41 (1.99–3.01)	2.40 (1.90–2.69)	0.334
<3.12	190 (34.99%)	42 (46.15%)	148 (32.74%)	0.027
≥3.12	49 (9.02%)	4 (4.40%)	45 (9.96%)	
Glucose, mmol/L	5.56 (4.88–6.80)	5.55 (4.99–6.79)	5.62 (4.79–7.30)	0.706
<3.92	9 (1.66%)	5 (5.49%)	4 (0.88%)	0.008
3.92–6.16	235 (43.28%)	37 (40.66%)	198 (43.81%)	
>6.16	140 (25.78%)	24 (26.37%)	116 (25.66%)	
Red blood cell distribution width SD, fL	41.00 (38.30–44.00)	41.88 (38.15–44.43)	40.10 (38.50–41.75)	0.037
D-dimer, mg/L	0.38 (0.22–0.77)	0.38 (0.21–0.80)	0.39 (0.26–0.66)	0.368
Oxygen saturation, %	97.80 (96.00–99.00)	96.30 (94.00–98.45)	98.00 (96.00–99.00)	0.246
*Imaging features*				
One side ground-glass opacity	98 (18.05%)	18 (19.78%)	80 (17.70%)	0.021
Both sides ground-glass opacity	345 (63.54%)	57 (62.64%)	288 (63.72%)	0.039
Consolidation	12 (2.21%)	0 (0%)	12 (2.65%)	<0.001
Others	40 (7.37%)	5 (5.49%)	35 (7.74%)	0.053
Normal	48 (8.84%)	11 (12.09%)	37 (8.19%)	0.001

Data are median (IQR), n (%), or n/N (%). *p* values were calculated by Mann-Whitney U test, *χ*² test, or Fisher's exact test, as appropriate.

**Table 2:** Clinical characteristics and laboratory findings in different age of hypertensive and non-hypertensive COVID-19 patients.

Characteristics	Total	Elderly (> 60 yr)	Non- elderly (≤60 yr)	*p* value
*Patients with hypertension*	(*n* = 91)	(*n* = 17)	(*n* = 74)	
Disease severity				
Mild	16 (17.58%)	2 (11.76%)	14 (18.92%)	0.050
General	68 (74.73%)	11 (64.71%)	57 (77.03%)	
Severe	7 (7.69%)	4 (23.53%)	3 (4.05%)	
Laboratory findings				
Triglycerides, mmol/L	1.21 (0.81–2.01)	1.50 (1.01–2.17)	0.79 (0.71–1.00)	0.005
Total bilirubin, umol/L	12.30 (8.86–16.95)	13.25 (9.54–17.28)	6.00 (4.74–11.65)	0.006
Indirect bilirubin, umol/L	8.00 (5.12–12.38)	8.50 (5.63–13.00)	6.90 (4.90–10.95)	0.009
< 1.7	64 (70.33%)	13 (76.47%)	51 (68.92%)	0.024
P <0.8, mmol/L	6 (6.60%)	2 (11.76%)	5 (6.76%)	0.028
Ca, mmol/L	2.23 (2.10–2.35)	2.07 (1.94–2.22)	2.25 (2.13–2.36)	0.013
Thrombin time> 16, s	17 (18.68%)	0 (0%)	17 (22.97%)	0.022
D-dimer, ug/ml	0.38 (0.20–0.79)	0.44 (0.28–1.36)	0.32 (0.19–0.57)	0.030
Red blood cell distribution width SD, fL	40.10 (38.50-41.75)	41.45 (39.43-47.20)	40.10 (36.80-41.00)	0.007
Red blood cell distribution width CV, %	12.30 (12.00-13.20)	12.95 (12.23-14.10)	12.20 (11.95-12.95)	0.035

*Patients without hypertension*	(*n* = 452)	(*n* = 82)	(*n* = 370)	
Disease severity				
Mild	51 (11.28%)	6 (7.32%)	45 (12.16%)	0.001
General	355 (78.54%)	58 (70.73%)	297 (80.27%)	
Severe	46 (10.18%)	18 (21.95%)	28 (7.57%)	
Laboratory findings				
Cholinesterase, IU/L	7454.00 (6121.25–8546.75)	7488.00 (5682.25–9250.25)	7435.00 (6256.25–8523.50)	0.018
Lactate dehydrogenase, IU/L	189.00 (156.67–233.10)	188.00 (146.50–220.46)	194.00(159.17–240.75)	0.016
Indirect bilirubin<1.7, umol/L	324 (71.68%)	56 (68.29%)	278 (75.14%)	0.005
*α*-L-fucosidase, U/L	26.00 (21.80-30.88)	28.00 (25.88-33.85)	25.00 (21.00-30.00)	0.032
*α*-Hydroxybutyrate dehydrogenase, IU/L	160.00 (137.28–197.96)	150.50 (122.0–180.22)	165.00(136.00–212.43)	0.01
Low-density lipoprotein, mmol/L	2.40 (1.99–3.01)	2.81 (2.20–3.37)	2.14 (2.08–2.28)	0.041
Total cholesterol, mmol/L	4.13 (3.59–4.85)	4.43 (3.83–5.08)	4.05 (3.53–4.79)	0.038
Complement C1q, mg/L	150.05 (140.14–166.21)	134.51 (125.51–136.09)	164.41(144.57–169.65)	0.028

**Table 3:** Clinical characteristics and laboratory findings in different gender of hypertensive and non-hypertensive COVID-19 patients.

Characteristics	Total	Male	Female	*p* value
*Patients with hypertension*	(*n* = 91)	(*n* = 51)	(*n* = 40)	
Disease severity				
Mild	16 (17.58%)	3 (5.88%)	13 (32.50%)	0.06
General	68 (74.73%)	46 (90.20%)	22 (55.00%)	
Severe	7 (7.69%)	2 (3.92%)	5 (12.50%)	
Symptoms				
Fever	39 (42.86%)	27 (52.94%)	12 (30.00%)	0.04
Laboratory findings				
Hematocrit, %	35.15 (13.34–42.78)	38.48 (17.01–50.67)	34.42 (5.11–45.67)	0.032
Total bile acid, umol/L	3.65 (2.30–7.23)	2.75 (1.58–5.13)	5.30 (3.05–7.98)	0.004
Total protein, g/L	66.40 (31.00–72.23)	68.20 (63.70–75.20)	62.90 (58.00–70.20)	0.005
< 60	14 (15.38%)	4 (7.84%)	10 (25.00%)	0.029
Globulin, g/L	27.00 (24.30–30.45)	28.30 (25.59–31.50)	26.00 (22.00–28.61)	0.011
Glucose, mmol/L	5.62 (4.79–7.30)	6.00 (4.85–8.47)	5.04 (4.67–6.20)	0.021
Cl, mmol/L	102.05 (99.65–105.00)	101.10 (99.06–103.85)	103.16 (100.39–105.28)	0.033
Fibrinogen, g/L	3.31 (2.68–4.03)	3.40 (2.97–4.55)	2.69 (2.29–3.54)	0.018

*Patients without hypertension*	(*n* = 452)	(*n* = 225)	(*n* = 227)	
Laboratory findings				
5‘nuclease, U/L	3.00 (2.25–6.40)	3.03 (2.17–6.72)	4.20 (3.00–7.60)	0.044
Total bilirubin<5.13, umol/L	43 (9.51%)	14 (6.22%)	28 (12.33%)	0.038
Urea<3.2, mmol/L	94 (20.80%)	39 (17.33%)	55 (24.23%)	0.011
Cystatin C, mg/L	1.00 (0.33–1.21)	1.10 (0.37–1.30)	0.97 (0.29–1.15)	0.010
Urea/creatinine	66.26 (47.20–78.86)	52.68 (24.97–69.79)	75.10 (53.24–96.09)	0.021
Glomerular filtration rate	110.31 (101.15–121.21)	106.53 (99.33–122.52)	115.70 (101.14–127.63)	0.010
Eosinophil count, ×109 per L				
< 0.05	182 (40.27%)	82 (36.44%)	100 (44.05%)	0.037
0.05–0.3	155 (34.29%)	86 (38.22%)	69 (30.40%)	
> 0.3	3 (0.66%)	0 (0%)	3 (1.32%)	
Creatinine, umol/L	62.00 (52.28–75.00)	66.85 (56.49–78.00)	61.81 (51.95–75.00)	<0.001
< 53	98 (21.68%)	33 (14.67%)	65 (28.63%)	<0.001
53–97	266 (58.85%)	142 (63.11%)	124 (54.63%)	
> 97	19 (4.20%)	11 (4.89%)	5 (2.20%)	
Red blood cell distribution width SD, fL				
< 35	9 (1.99%)	8 (3.56%)	1 (0.44%)	0.011
35–56	325 (71.90%)	158 (70.22%)	167 (73.57%)	
> 56	10 (2.21%)	2 (8.89%)	8 (3.52%)	

**Table 4:** Risk factors associated with COVID-19 patients with hypertension.

	Univariable OR (95% CI)	*p* value	Multivariable OR (95% CI)	*p* value
Demographics				
Age	1.05 (1.03–1.07)	0.121	1.04 (1.00–1.19)	<0.001
Comorbidities				
Diabetes	3.734 (1.965–7.096)	<0.001	3.112 (1.339–6.924)	0.005
Coronary heart disease	8.159 (2.828–23.537)	<0.001	8.815 (2.574–30.189)	0.001
Cerebral vascular disease	4.11 (1.082–15.614)	0.038		
Laboratory findings				
Globulin, g/L				
< 20	3.503 (0.625–19.64)	0.154		
20–30	1 (ref)	—		
> 30	—	—		
Triglyceride, mmol/L				
0.24–1.86	1 (ref)	—		
> 1.86	1.948 (0.664–2.355)	0.771		
High density lipoprotein, mmol/L				
< 1.03	107.209 (1.48–127.81)	0.033	123.67 (1.39–137.94)	<0.001
1.03–1.55	1 (ref)	—		
> 1.55	0.581 (0.04–8.501)	0.692		
Neutrophil count, ×10^9^ per L	0.994 (0.63–1.081)	0.09		
Lymphocyte count, ×10^9^ per L	0.982 (0.966–1.998)	0.032	0.797 (0.628–1.899)	0.033
<0.8	1.146 (0.023–1.937)	0.043	2.532 (0.987–2.900)	0.028
0.8–4	1 (ref)	—		
>4	—	—		
Lymphocyte percentage, %				
< 20	0.928 (0.863–0.998)	0.043		
20–40	1 (ref)	—		
>40	—	—		
Monocyte count, ×10^9^ per L	1.501 (1.035–2.175)	0.032		
Mean hemoglobin, pg				
< 27	0.935 (0.742–1.178)	0.57		
27–31	1 (ref)	—		
> 31	0.645 (0.392–1.061)	0.084		
Basophil percentage, ×10^9^ per L	0.327 (0.094–1.136)	0.079		
Haemoglobin, g/L				
< 115	1.064 (0.99–1.144)	0.093		
115–150	1 (ref)	—		
> 150	0.924 (0.837–1.02)	0.119		
Red blood cell distribution width SD, fL	0.937 (0.889–0.998)	0.015	0.851 (0.793–0.913)	<0.001
Thrombin time, s	1.121 (0.993–1.265)	0.064	1.507 (1.280–1.774)	<0.001
Glucose	7.03 (1.87–22.30)	0.0079	4.85 (0.76–39.81)	0.049
D-dimer, mg/L	1.768 (0.876–6.77)	0.089	2.111 (0.56–8.796)	0.05

**Table 5:** Risk factors associated with elderly COVID-19 patients with hypertension.

	Univariable OR (95% CI)	*p* value	Multivariable OR (95% CI)	*p* value
Total bilirubin, umol/L	1.912 (0.832–1.999)	0.057	1.014 (0.230–1.05)	0.043
< 5.13	—	—		
5.13–22.24	1 (ref)	—		
> 22.24	1.83 (0.498–2.387)	0.476		
Direct bilirubin, umol/L				
> 6.8	0.785 (0.596–1.036)	0.087		
Indirect bilirubin, umol/L	0.891 (0.791–1.003)	0.056		
< 1.7	—	—		
1.7–10.2	1 (ref)	—		
> 10.2	0.922 (0.72–1.168)	0.617		
Triglyceride>1.86, mmol/L	1.208 (0.051–1.853)	0.029	1.173 (0.049–1.617)	0.007
Ca, mmol/L	0.004 (0.002–0.441)	0.022		
Creatinine, umol/L	1.062 (0.918–1.23)	0.418		
White blood cell count, ×10^9^ per L	1.028 (0.027–39.163)	0.088		
Red blood cell distribution width SD, fL	1.914 (0.627–2.333)	0.048		

**Table 6:** Risk factors associated with male COVID-19 patients with hypertension.

	Univariable OR (95% CI)	*p* value	Multivariable OR (95% CI)	*p* value
Albumin, g/L				
< 35	1.478 (0.955–2.288)	0.08		
35–51	1 (ref)	—		
> 51	—	—		
High density lipoprotein, mmol/L				
< 1.03	67.863 (1.021–436.457)	0.049	61.977 (0.656–679.885)	0.039
1.03–1.55	1 (ref)	—		
> 1.55	2.684 (0.002–3082.751)	0.784		
Creatinine, umol/L	1.982 (0.962–2.003)	0.087	1.223 (1.055–1.658)	0.032
Carbon dioxide, mmol/L	1.14 (0.989–1.313)	0.071		
Neutrophil count, ×10^9^ per L	1.04 (0.993–1.089)	0.094		
Lymphocyte percentage, %	0.967 (0.938–0.997)	0.029	0.977 (0.659–2.085)	0.034
< 20	0.885 (0.808–0.97)	0.009		
20–40	1 (ref)	—		
>40	0.672 (0.26–1.734)	0.411		
Basophil percentage, ×10^9^ per L	0.128 (0.022–0.733)	0.021	0.112 (0.014–0.907)	0.041
≤ 1	0.155 (0.024–0.984)	0.048		
> 1	—	—		
Mean hemoglobin, pg				
< 27	1.382 (0.92–2.412)	0.255		
27–31	1 (ref)	—		
> 31	0.452 (0.183–1.117)	0.086		
Thrombin time, s				
< 16	0.991 (0.538–1.827)	0.978		
16–18	1 (ref)	—		
> 18	1.167 (0.731–1.865)	0.118		
Red blood cell distribution width SD, fL	0.947 (0.889–1.008)	0.09		
Fibrinogen, g/L	1.398 (0.987–1.997)	0.066	1.598 (0.803–2.787)	0.044

**Table 7:** Risk factors associated with female COVID-19 patients with hypertension.

	Univariable OR (95% CI)	*p* Value	Multivariable OR (95% CI)	*p* Value
Alanine aminotransferase, IU/L	0.98 (0.957–1.003)	0.092		
Aspartate aminotransferase, IU/L	0.949 (0.906–0.995)	0.031		
Total bile acid, umol/L				
<0.1	0.852 (0.534–1.361)	0.033		
0.1–10	1 (ref)	–		
>10	1.895 (0.568–3.308)	0.033	4.611 (0.469–7.778)	0.029
Total protein, g/L	0.955 (0.916–0.996)	0.03	0.677 (0.544–0.990)	0.036
Globulin, g/L	0.91 (0.846–0.98)	0.012		
Total bilirubin, umol/L				
<5.13	1.08(0.417–2.789)	0.874		
5.13–22.24	1 (ref)	–		
>22.24	1.06 (0.99–1.136)	0.094		
Indirect bilirubin, umol/L	1.033 (0.996–1.073)	0.084		
Low-density lipoprotein, mmol/L	0.552 (0.282–1.083)	0.084	0.319 (0.117–0.872)	0.026
Urea/Creatinine	0.976 (0.956–0.996)	0.019		
Glucose, mmol/L	0.743 (0.05–1.002)	0.052		
Lymphocyte count, ×10^9^ per L	0.897 (0.809–1.699)	0.059		
<0.8	1.071 (0.003–1.616)	0.097		
0.8–4	1 (ref)	–		
>4	–	–		
Monocyte count, ×10^9^ per L	1.472 (0.989–2.19)	0.057		
Red blood cell distribution width SD, fL	0.915 (0.828–1.012)	0.083		
<35	0.362 (0.177–0.742)	0.005		
35–56	1 (ref)	–		
>56	0.634 (0.279–1.438)	0.275		
Mean hemoglobin, pg	0.645 (0.422–0.985)	0.042	0.905 (0.872–0.939)	0.037
Thrombin time>18, s	1.748 (0.95–3.216)	0.073		
Fibrinogen, g/L	0.603 (0.342–1.061)	0.079		

## Data Availability

All data were included in the manuscript.

## Supplementary Materials

Supplementary Materials It shows the laboratory characteristics of COVID-19 patients with and without hypertension.
