# Supplementary material for: Retrospective Study of Aging and Sex-Specific Risk Factors of COVID-19 with Hypertension in China
Source: Cardiovasc Ther. 2022 Jun 28;2022:5978314. doi: 10.1155/2022/5978314 (PMC9240958; doi:10.1155/2022/5978314)
Supplement: Supplementary Materials — It shows the laboratory characteristics of COVID-19 patients with and without hypertension. [file 5978314.f1.docx]

| SUPPLEMENTARY TABLE 1:Laboratory characteristics of COVID-19 patients with and without hypertension | | | | |
| --- | --- | --- | --- | --- |
|  | Total (*n*=543) | Patients with hypertension (*n*=91) | Patients without hypertension (*n*=452) | *p* value |
| **Demographics** |  |  |  |  |
| Age, years | 47.00 (34.00‒57.00) | 47.00 (30.00‒57.00) | 47.00 (35.00‒57.00) | 0.633 |
| >60 | 99 (18.23%) | 17 (18.68%) | 82 (18.14%) | 0.903 |
| ≤60 | 444 (81.77%) | 74 (81.32%) | 370 (81.86%) |  |
| Sex |  |  |  |  |
| Male | 267 (49.17%) | 40 (43.96%) | 227 (50.22%) | 0.276 |
| Female | 276 (50.83%) | 51 (56.04%) | 225 (49.78%) |  |
| **Disease severity** |  |  |  |  |
| Mild | 67 (12.34%) | 16 (17.58%) | 51 (11.28%) | 0.102 |
| General | 423 (77.90%) | 68 (74.73%) | 355 (78.54%) |  |
| Severe | 53 (9.76%) | 7 (7.69%) | 46 (10.18%) |  |
| **Comorbidity** |  |  |  |  |
| Diabetes | 46 (8.47%) | 18 (19.78%) | 28 (6.19%) | <0.0001 |
| Coronary heart disease | 15 (2.76%) | 9 (9.89%) | 6 (1.32%) | <0.0001 |
| Cerebral vascular disease | 9 (1.66%) | 4 (4.40%) | 5 (1.11%) | 0.025 |
| **Medication information** |  |  |  |  |
| Antiviral drugs | 450 (82.87%) | 75 (82.42%) | 375 (82.96%) | 0.900 |
| Antibiotic | 245 (45.12%) | 41 (45.05%) | 204 (45.13%) | 0.989 |
| Infusion | 42 (7.73%) | 6 (6.59%) | 36 (7.96%) | 0.655 |
| Traditional Chinese medicine | 530 (97.61%) | 88 (96.70%) | 442 (97.79%) | 0.537 |
| **Outcome** |  |  |  |  |
| Disease duration,days | 16.00 (12.00‒21.00) | 17.00 (13.00‒21.00) | 16.00 (12.00‒21.00) | 0.632 |
| **Symptoms** |  |  |  |  |
| Cough | 273 (50.28%) | 53 (58.24%) | 220 (48.67%) | 0.096 |
| Fever | 221 (40.70%) | 39 (42.86%) | 182 (40.27%) | 0.646 |
| Dry cough | 173 (31.86%) | 30 (32.97%) | 143 (31.64%) | 0.804 |
| Fatigue | 106 (19.52%) | 20 (21.98%) | 86 (19.03%) | 0.517 |
| Anorexia | 81 (14.91%) | 11 (12.09%) | 70 (15.49%) | 0.407 |
| Asthma | 65 (11.97%) | 7 (7.69%) | 58 (12.83%) | 0.169 |
| Diarrhea | 59 (10.87%) | 8 (8.79%) | 51 (11.28%) | 0.486 |
| Sore throat | 33 (6.08%) | 4 (4.40%) | 29 (6.42%) | 0.462 |
| Chills | 19 (3.50%) | 4 (4.40%) | 15 (3.31%) | 0.61 |
| Nasal congestion | 18 (3.31%) | 4 (4.40%) | 14 (3.10%) | 0.528 |
| Runny nose | 17 (3.13%) | 5 (5.49%) | 12 (2.65%) | 0.156 |
| **Laboratory findings** |  |  |  |  |
| Alanine aminotransferase,IU/L | 24.00 (16.35‒39.50) | 24.00 (17.00‒41.50) | 24.00 (14.4‒-25.83) | 0.268 |
| ＜7 | 6 (0.92%) | 1 (1.10%) | 5 (1.11%) | 0.264 |
| 7‒40 | 318 (58.56%) | 57 (62.64%) | 261 (57.74%) |  |
| ＞40 | 105 (19.34%) | 14 (15.38%) | 91 (20.13%) |  |
| Aspartate aminotransferase,IU/L | 22.00 (17.00‒31.00) | 22.00 (17.00‒31.95) | 19.15 (16.00‒29.75) | 0.324 |
| ＜12 | 15 (2.76%) | 4 (4.40%) | 11 (2.43%) | 0.09 |
| 12‒35 | 349 (64.27%) | 61 (67.03%) | 288 (63.72%) |  |
| ＞35 | 64 (11.79%) | 7 (7.69%) | 57 (12.61%) |  |
| γ-glutamyl transpeptidase,IU/L | 30.24 (19.00‒58.00) | 30.00 (19.00‒57.44) | 33.00 (21.00‒59.00) | 0.55 |
| ＜7 | 0 (0%) | 0 (0%) | 0 (0%) | 0.729 |
| 7-45 | 277 (51.01%) | 45 (49.45%) | 232 (51.33%) |  |
| ＞45 | 148 (27.26%) | 26 (28.57%) | 122 (26.99%) |  |
| Alkaline phosphatase,IU/L | 66.60 (51.00‒86.90) | 67.00 (51.00‒88.63) | 64.00 (48.00‒78.25) | 0.095 |
| ＜50 | 96 (17.68%) | 22 (24.18%) | 74 (16.37%) | 0.181 |
| 50‒75 | 163 (30.01%) | 26(28.57%) | 137(30.31%) |  |
| ＞75 | 165 (30.39%) | 26(28.57%) | 139(30.75%) |  |
| Cholinesterase,IU/L | 7445.00 (6205.00‒8540.00) | 7407.00(6281.00‒8467.00) | 7454.00(6121.25‒8546.75) | 0.506 |
| ＜4000 | 8 (1.47%) | 2(2.20%) | 6(1.33%) | 0.737 |
| 4000‒12000 | 206 (37.94%) | 40(43.96%) | 166(36.73%) |  |
| ＞12000 | 1 (0.18%) | 1(1.10%) | 0(0%) |  |
| Lactate dehydrogenase,IU/L | 209.40 (87.65-289.90) | 209.52(88.63-300.02) | 208.82(83.11-297.77) | 0.955 |
| 20‒240 | 290 (53.41%) | 45(49.45%) | 245(54.20%) | 0.497 |
| ＞240 | 86 (15.84%) | 16(17.58%) | 70(15.49%) |  |
| α-hydroxybutyrate dehydrogenase,IU/L | 157.77 (133.75‒200.65) | 145.50(123.75‒245.50) | 159.77(134.14‒200.09) | 0.673 |
| ＜72 | 3 (0.55%) | 0(0%) | 3(0.66%) | 0.438 |
| 72‒182 | 108 (19.89%) | 18(19.78%) | 90(19.91%) |  |
| ＞182 | 55 (10.13%) | 11(12.09%) | 44(9.73%) |  |
| ApoA-1,g/L | 0.95 (0.76‒1.11) | 0.95(0.72-1.15) | 1.01(0.89‒1.10) | 0.313 |
| ＜1 | 58 (10.68%) | 10(10.99%) | 48(10.62%) | 0.384 |
| 1‒1.6 | 45 (8.29%) | 11(12.09%) | 34(7.52%) |  |
| ＞1.6g/L | 1 (0.18%) | 0(0%) | 1(0.22%) |  |
| Apo-B,g/L | 0.82 (0.61‒1.02) | 0.82(0.60‒1.03) | 0.87(0.66‒0.95) | 0.79 |
| ＜0.6g/L | 22 (4.05%) | 2(2.20%) | 20(4.42%) | 0.856 |
| 0.6‒1.1g/L | 65 (11.97%) | 16(17.58%) | 49(10.84%) |  |
| ＞1.1g/L | 16 (2.95%) | 2(2.20%) | 14(3.10%) |  |
| Total bile acid,umol/L | 3.80 (2.60‒6.20) | 3.80(2.60‒6.10) | 3.65(2.30‒7.23) | 0.726 |
| ＜0.1 | 0 (0%) | 0(0%) | 0(0%) | 0.406 |
| 0.1‒10 | 303 (55.80%) | 52(57.14%) | 251(55.53%) |  |
| ＞10 | 20 (3.72%) | 2(2.20%) | 18(3.98%) |  |
| Total protein,g/L | 67.00 (61.90‒73.60) | 67.30(62.00‒73.82) | 66.40(31.00‒72.23) | 0.535 |
| ＜60 | 82 (15.10%) | 14(15.38%) | 68(15.04%) | 0.555 |
| 60‒80 | 366 (67.40%) | 63(69.23%) | 303(67.04%) |  |
| ＞80 | 31 (5.71%) | 3(3.30%) | 28(6.19%) |  |
| Albumin,g/L | 39.52 (35.57‒43.93) | 39.52(35.88‒44.00) | 39.50(35.03‒42.95) | 0.745 |
| ＜35 | 101 (18.60%) | 18(19.78%) | 83(18.36%) | 0.854 |
| 35‒51 | 372 (68.51%) | 61(67.03%) | 311(68.81%) |  |
| ＞51 | 9 (1.66%) | 1(1.10%) | 8(1.77%) |  |
| Globulin,g/L | 28.00 (25.00‒31.00) | 28.06(25.13‒31.06) | 27.00(24.30‒30.45) | 0.334 |
| ＜20 | 22 (4.05%) | 4(4.40%) | 18(3.98%) | 0.787 |
| 20‒30 | 305 (56.17%) | 53(58.24%) | 252(55.75%) |  |
| ＞30 | 154 (28.36%) | 23(25.27%) | 131(28.98%) |  |
| A/G | 1.42 (1.26‒1.63) | 1.42(1.26‒1.63) | 1.47(1.22‒1.68) | 0.905 |
| Prealbumin,mg/L | 194.70 (122.50‒251.00) | 193.98(122.50‒254.08) | 193.98(122.50‒254.08) | 0.821 |
| ＜280 | 244 (44.94%) | 43(47.25%) | 201(44.47%) | 0.586 |
| 280‒360 | 28 (5.16%) | 5(5.50%) | 23(5.09%) |  |
| >360 | 5 (0.92%) | 0(0%) | 5(1.10%) |  |
| Ischemia modified albumin,U/ml | 75.67 (69.66‒80.70) | 72.98(69.31‒80.52) | 80.18(72.83‒83.09) | 0.248 |
| ＜85 | 19 (3.50%) | 4(4.40%) | 15(3.32%) | 0.419 |
| ＞85 | 2 (0.37%) | 1(1.10%) | 1(0.22%) |  |
| Total bilirubin,umol/L | 11.60 (7.15‒16.70) | 11.50(7.05‒16.66) | 12.30(8.86‒16.95) | 0.742 |
| ＜5.13 | 52 (9.58%) | 10(10.99%) | 42(9.29%) | 0.626 |
| 5.1322.24 | 360 (66.30%) | 57(62.64%) | 303(67.04%) |  |
| ＞22.24 | 65 (11.97%) | 13(14.29%) | 52(11.50%) |  |
| Direct bilirubin, umol/L | 3.55 (2.20-5.50) | 3.40(2.20‒5.58) | 3.80(2.33‒5.19) | 0.87 |
| ≤6.8 | 389 (71.64%) | 65(71.43%) | 324(71.68%) | 0.953 |
| >6.8 | 67 (12.33%) | 11(12.09%) | 56(12.39%) |  |
| Indirect bilirubin,umol/L | 7.50 (4.60‒11.40) | 8.73(6.31‒12.08) | 10.19(9.73‒10.97) | 0.097 |
| ＜1.7 | 16 (2.95%) | 3(3.30%) | 13(2.88%) | 0.661 |
| 1.7‒10.2 | 309 (56.91%) | 48(52.75%) | 261(57.74%) |  |
| ＞10.2 | 132 (24.31%) | 25(27.47%) | 107(23.67%) |  |
| Low-density lipoprotein,mmol/L | 2.40 (1.96‒2.94) | 2.41(1.99‒3.01) | 2.40(1.90‒2.69) | 0.334 |
| ＜3.12 | 190 (34.99%) | 42(46.15%) | 148(32.74%) | 0.027 |
| ≥3.12 | 49 (9.02%) | 4(4.40%) | 45(9.96%) |  |
| High density lipoprotein,mmol/L | 1.04 (0.86‒1.25) | 1.05(0.84‒1.25) | 1.02(0.90‒1.24) | 0.89 |
| ＜1.03 | 117 (21.55%) | 24(26.37%) | 93(20.58%) | 0.898 |
| 1.03‒1.55 | 100 (18.41%) | 19(20.88%) | 81(17.92%) |  |
| ＞1.55 | 24 (4.42%) | 4(4.40%) | 20(4.42%) |  |
| Total cholesterol, mmol/L | 4.12 (3.59‒4.83) | 4.13(3.59‒4.85) | 4.10(3.55‒4.71) | 0.095 |
| ＜2.8 | 18 (3.31%) | 4(4.40%) | 14(3.10%) | 0.899 |
| 2.8‒5.2 | 266 (48.99%) | 50(54.95%) | 216(47.79%) |  |
| ＞5.2 | 52 (9.58%) | 9(9.89%) | 43(9.51%) |  |
| Creatine kinase,IU/L | 55.00 (37.00‒84.00) | 54.04(37.00‒83.75) | 56.29(36.73‒89.00) | 0.743 |
| ＜25 | 33 (6.08%) | 3(3.30%) | 30(6.64%) | 0.778 |
| 25‒190 | 305 (56.17%) | 52(57.14%) | 253(55.97%) |  |
| ＞190 | 29 (5.34%) | 2(2.20%) | 17(3.76%) |  |
| Amylase,U/L | 42.31 (54.00‒67.45) | 55.00(43.00‒67.35) | 50.00(39.31‒71.50) | 0.66 |
| ＜220 | 175 (32.23%) | 28(30.77%) | 147(32.52%) | 0.51 |
| Lipase,U/L | 39.70 (29.65‒50.40) | 39.75(30.05‒51.33) | 35.20(25.00‒47.08) | 0.6 |
| ＜60 | 60 (11.05%) | 11(12.09%) | 49(10.84%) | 0.487 |
| ＞60 | 5 (0.92%) | 0(0%) | 5(1.11%) |  |
| Adenosine deaminase,IU/L | 11.70 (9.00‒14.33) | 11.35(9.02‒14.84) | 12.28(9.47‒14.00) | 0.575 |
| ＜4Iu/L | 1 (0.18%) | 0(0%) | 1(0.22%) | 0.542 |
| 4‒20Iu/L | 159 (29.28%) | 31(34.07%) | 128(28.32%) |  |
| ＞20Iu/L | 5 (0.92%) | 2(2.20%) | 3(0.66%) |  |
| Triglyceride, mmol/L | 1.40 (0.96‒2.05) | 1.75(1.27‒2.53) | 1.60(0.51‒1.98) | 0.538 |
| 0.24‒1.86 | 214 (39.41%) | 40(43.96%) | 174(38.5%) | 0.453 |
| ＞1.86 | 98 (18.05%) | 19(20.88%) | 79(17.48%) |  |
| Triacylglycerol, mmol/L | 1.43 (0.88‒2.27) | 1.36(0.73‒2.40) | 1.77(1.03‒2.23) | 0.638 |
| ＜1.7 | 9 (1.66%) | 1(1.10%) | 8(1.77%) | 0.998 |
| ＞1.7 | 7 (1.3%) | 2(2.20%) | 5(1.11%) |  |
| Urea, mmol/L | 3.90 (3.10‒5.00) | 3.96(3.13‒5.00) | 3.80(2.88‒4.85) | 0.413 |
| ＜3.2 | 116 (21.36%) | 22(24.18%) | 94(20.80%) | 0.764 |
| 3.2‒7.1 | 276 (50.83%) | 44(48.35%) | 232(51.33%) |  |
| ＞7.1 | 23 (4.24%) | 4(4.40%) | 19(4.20%) |  |
| Uric acid,umol/L | 278.94 (214.00‒350.85) | 280.00(216.00‒357.00) | 268.63(191.75‒340.48) | 0.132 |
| ＜140 | 27 (4.97%) | 6(6.60%) | 21(4.65%) | 0.455 |
| 140‒420 | 374 (68.88%) | 62(68.13%) | 312(69.03%) |  |
| ＞420 | 52 (9.58%) | 6(6.60%) | 46(10.18%) |  |
| Creatinine, umol/L | 62.00 (52.00‒75.15) | 62.00(52.28‒75.00) | 61.10(48.00‒76.40) | 0.32 |
| ＜53 | 124 (22.84%) | 26(28.57%) | 98(21.68%) | 0.105 |
| 53‒97 | 313 (57.64%) | 47(51.65%) | 266(58.85%) |  |
| ＞97 | 18 (3.31%) | 2(2.20%) | 16(3.54%) |  |
| Complement C1q,mg/L | 157.20 (143.16‒166.53) | 150.05(140.14‒166.21) | 166.15(146.1‒172.44) | 0.313 |
| ＜157 | 8 (1.47%) | 1(1.10%) | 7(1.55%) | 0.554 |
| 157‒237 | 8 (1.47%) | 2(2.20%) | 6(1.33%) |  |
| α-L-fucosidase,U/L | 26.00 (21.37‒29.98) | 26.00(21.80‒30.88) | 25.50(19.50‒28.60) | 0.402 |
| ＜40 | 107 (19.71%) | 19(20.88%) | 88(19.47%) | 0.616 |
| ＞40 | 9 (1.66%) | 1(1.10%) | 8(1.77%) |  |
| 5‘nuclease, U/L | 3.00 (2.38‒5.73) | 3.00(2.25‒6.40) | 3.20(2.50‒4.50) | 0.865 |
| ＜10 | 29 (5.34%) | 5(5.50%) | 24(5.31%) | 0.663 |
| ＞10 | 1 (0.18%) | 0(0%) | 1(0.22%) |  |
| Cystatin C, mg/L | 0.94 (0.80‒1.13) | 1.00 (0.33‒1.23） | 0.93 (0.21‒1.35） | 0.240 |
| ＜0.54 | 2 (0.37%) | 0(0%) | 2(0.44%) | 0.012 |
| 0.54‒1.5 | 181 (33.33%) | 34(37.36%) | 147(32.52%) |  |
| ＞1.5 | 11 (2.03%) | 0(0%) | 11(2.43%) |  |
| Urea/Creatinine | 61.63 (5.55‒90.47) | 69.89(38.16‒96.09) | 44.14(0.07‒68.36) | 0.024 |
| Glomerular filtration rate | 111.25 (102.37-120.79) | 110.31(101.15‒121.21) | 111.62(103.73‒119.71) | 0.759 |
| ＜90 | 6 (1.10%) | 0 (0%) | 6 (1.33%） | 0.013 |
| ≥90 | 63 (11.60%) | 10 (10.99%) | 53 (11.73%） |  |
| Glucose,mmol/L | 5.56 (4.88-6.80) | 5.55(4.99‒6.79) | 5.62(4.79‒7.30) | 0.706 |
| ＜3.92 | 9 (1.66%) | 5(5.49%) | 4(0.88%) | 0.008 |
| 3.92‒6.16 | 235 (43.28%) | 37(40.66%) | 198(43.81%) |  |
| ＞6.16 | 140 (25.78%) | 24(26.37%) | 116(25.66%) |  |
| kmmol/L(3.5-5.1) | 3.90 (3.59‒4.20) | 3.95(0.51‒4.65) | 3.80(3.51‒4.19) | 0.194 |
| ＜3.5 | 72 (13.26%) | 14(15.38%) | 58(12.83%) | 0.361 |
| 3.5‒5.1 | 371 (68.32%) | 63(69.23%) | 308(68.14%) |  |
| ＞5.1 | 7 (1.29%) | 0(0%) | 7(1.55%) |  |
| Na,mmol/L | 139.00 (137.00‒140.95) | 139.00(137.00‒140.80) | 139.20(137.00‒141.30) | 0.783 |
| ＜136 | 100 (18.41%) | 16(17.58%) | 84(18.58%) | 0.886 |
| 136‒146 | 337 (62.06%) | 58(63.74%) | 279(61.83%) |  |
| ＞146 | 8 (1.47%) | 1(1.10%) | 7(1.55%) |  |
| Ca, mmol/L | 2.24 (2.12‒2.36) | 2.24(2.21‒2.36) | 2.23(2.10‒2.35) | 0.574 |
| ＜2.1 | 87 (16.02%) | 16(17.58%) | 71(15.71%) | 0.489 |
| 2.1‒2.8 | 327 (60.22%) | 56(61.54%) | 271(59.96%) |  |
| ＞2.8 | 7 (1.29%) | 0(0%) | 7(1.55%) |  |
| Cl,mmol/L | 102.80 (100.00‒105.03) | 103.00(100.08‒105.00) | 102.05(99.65‒105.00) | 0.193 |
| ＜96 | 25 (4.60%) | 3(3.30%) | 22(4.87%) | 0.704 |
| 96‒106 | 353 (65.01%) | 59(64.84%) | 294(65.04%) |  |
| ＞106 | 62 (11.42%) | 12(13.19%) | 50(11.06%) |  |
| C02, mmol/L | 25.00 (23.80‒26.91) | 25.00(23.38‒26.90) | 25.90(24.00‒27.00) | 0.224 |
| ＜23 | 45 (8.29%) | 5(5.50%) | 40(8.85%) | 0.338 |
| 23‒31 | 225 (41.44%) | 42(46.15%) | 183(40.49%) |  |
| ＞31 | 11 (2.03%) | 3(3.30%) | 8(1.77%) |  |
| P, mmol/L | 1.04 (0.88‒1.23) | 1.06(0.88‒1.24) | 0.99(0.89‒1.16) | 0.194 |
| ＜0.8 | 28 (5.16%) | 6(6.60%) | 22(4.87%) | 0.67 |
| 0.8‒1.61 | 168 (30.94%) | 28(30.77%) | 140(30.97%) |  |
| ＞1.61 | 2 (0.37%) | 0(0%) | 2(0.44%) |  |
| Total osmotic pressure, mOSM/l | 295.10 (290.61‒299.03) | 295.40(290.92‒299.03) | 292.40(287.25‒299.60) | 0.299 |
| ＜280 | 4 (0.74%) | 1(1.10%) | 3(0.66%) | 0.296 |
| 280‒310 | 79 (14.55%) | 11(12.09%) | 51(11.28%) |  |
| Blood lactic acid, mmol/L | 2.90 (2.45‒2.99) | 2.77 (2.04‒2.89) | 2.85(2.45‒3.07) | 0.432 |
| Anion gap,mmol/L | 10.70 (9.00‒12.20) | 10.85(9.00‒12.15) | 10.00(8.00‒12.30) | 0.338 |
| ＜8 | 11 (2.03%) | 2(2.20%) | 9(1.99%) | 0.733 |
| 8‒16 | 78 (14.36%) | 13(14.29%) | 65(14.38%) |  |
| ＞16 | 3 (0.55%) | 0(0%) | 3(0.66%) |  |
| White blood cell count, 10^9^/L | 5.90 (4.51‒7.54) | 5.80(4.42‒7.43) | 6.60(5.03‒8.10) | 0.364 |
| ＜4 | 62 (11.42%) | 5(5.50%) | 57(12.61%) | 0.13 |
| 4‒10 | 375 (69.06%) | 68(74.73%) | 307(67.92%) |  |
| ＞10 | 42 (7.73%) | 6(6.60%) | 36(7.96%) |  |
| Neutrophil count,10^9/L | 3.71 (2.78‒5.31) | 3.66(2.69‒5.22) | 4.40(3.00‒6.19) | 0.234 |
| ＜1.8 | 35 (6.45%) | 3(3.30%) | 33(7.30%) | 0.032 |
| 1.8‒6.3 | 366 (67.40%) | 57(62.64%) | 309(68.36%) |  |
| ＞6.3 | 76 (14.00%) | 18(19.78%) | 58(12.83%) |  |
| Neutrophil percentage, % | 65.70 (56.98‒75.18) | 65.30(56.60‒74.70) | 68.80(57.90‒78.00) | 0.12 |
| ＜40 | 15 (2.76%) | 2(2.20%) | 13(2.88%) | 0.248 |
| 40‒75 | 344 (63.35%) | 53(58.24%) | 291(64.38%) |  |
| ＞75 | 121 (22.28%) | 24(26.37%) | 97(21.46%) |  |
| Lymphocyte count, 10^9/L | 1.38 (0.95‒1.80) | 1.37(0.96‒1.78) | 1.50(0.89‒1.94) | 0.175 |
| ＜0.8 | 82 (15.10%) | 17(18.68%) | 65(14.38%) | 0.048 |
| 0.8‒4 | 398 (73.30%) | 61(67.03%) | 337(74.56%) |  |
| ＞4 | 7 (1.29%) | 3(3.30%) | 4(0.88%) |  |
| Lymphocyte percentage, % | 24.25 (16.80‒32.70) | 24.30(17.10‒33.00) | 22.10(14.40‒31.35) | 0.053 |
| ＜20 | 172 (31.68%) | 33(36.26%) | 139(30.75%) | 0.156 |
| 20‒40 | 273 (50.28%) | 45(49.45%) | 228(50.44%) |  |
| ＞40 | 43 (7.99%) | 3(3.30%) | 40(8.85%) |  |
| Monocyte count, 10^9/L | 0.43 (0.32‒0.56) | 0.42(0.32‒0.55) | 0.47(0.33‒0.59) | 0.126 |
| ＜0.12 | 10 (1.84%) | 2(2.20%) | 8(1.77%) | 0.377 |
| 0.12‒0.8 | 369 (67.96%) | 63(69.23%) | 306(67.70%) |  |
| ＞0.8 | 33 (6.08%) | 8(8.80%) | 25(5.53%) |  |
| Monocyte percentage% | 7.40 (5.90‒9.40) | 7.45(5.90‒9.50) | 7.00(5.40‒9.15) | 0.667 |
| ＜3 | 24 (4.42%) | 6(6.60%) | 18(3.98%) | 0.625 |
| 3‒8 | 215 (39.59%) | 37(40.66%) | 178(39.38%) |  |
| ＞8 | 174 (32.04%) | 30(32.97%) | 144(31.86%) |  |
| Eosinophil count, 10^9/L | 0.04 (0.01‒0.09) | 0.04(0.01‒0.09) | 0.03(0.01‒0.09) | 0.578 |
| ＜0.05 | 223 (41.07%) | 41(45.05%) | 182(40.27%) | 0.323 |
| 0.05‒0.3 | 184 (33.89%) | 29(31.87%) | 155(34.29%) |  |
| ＞0.3 | 5 (0.92%) | 2(2.20%) | 3(0.66%) |  |
| Eosinophil percentage, % | 0.60 (0.10‒1.60) | 0.60(0.08‒1.60) | 0.40(0.10‒1.60) | 0.567 |
| ＜1 | 252 (46.41%) | 45(49.45%) | 207(45.80%) | 0.666 |
| 1‒4 | 143 (26.34%) | 28(30.77%) | 115(25.44%) |  |
| ＞4 | 18 (3.31%) | 2(2.20%) | 16(3.54%) |  |
| Basophil count, 10^9/L | 0.01 (0.01‒0.02) | 0.01(0.01‒0.02) | 0.01(0.01‒0.03) | 0.704 |
| Basophil percentage, 10^9/L | 0.26 (0.10‒0.40) | 0.22(0.13‒0.36) | 0.25 (0.08‒0.40) | 0.037 |
| ＜1 | 401 (73.85%) | 73(80.22%) | 328(72.57%) | 0.005 |
| ≥1 | 5 (0.92%) | 0(0%) | 5(1.10%) |  |
| Red blood cell count,10^12/L | 4.46 (4.08‒4.86) | 4.46(4.07‒4.87) | 4.42(4.12‒4.86) | 0.992 |
| ＜3.8 | 62 (11.42%) | 11(12.09%) | 51(11.28%) | 0.998 |
| 3.8‒5.1 | 353 (65.01%) | 57(62.64%) | 296(65.49%) |  |
| ＞5.1 | 74 (13.63%) | 13(14.29%) | 61(13.50%) |  |
| Haemoglobin, g/L | 134.00 (123.07‒148.48) | 132.03(123.11‒147.15) | 135.78(123.04‒148.23) | 0.014 |
| ＜115 | 74 (13.63%) | 14(15.38%) | 60(13.27%) | 0.463 |
| 115‒150 | 305 (56.17%) | 50(54.95%) | 255(56.42%) |  |
| ＞150 | 102 (1878%) | 15(16.48%) | 87(19.25%) |  |
| Hematocrit, % | 39.50 (34.90‒43.90) | 37.99(26.83‒48.08) | 35.15(13.34‒44.77) | 0.356 |
| ＜37 | 119 (21.92%) | 22(24.18%) | 97(21.46%) | 0.463 |
| 37‒50 | 260 (47.88%) | 48(52.75%) | 212(46.90%) |  |
| ＞50 | 91 (16.76%) | 10(10.99%) | 81(17.92%) |  |
| Average volume of red blood cells,fl | 90.20 (87.00‒93.40) | 90.50(87.10‒93.90) | 89.70(86.85‒92.65) | 0.138 |
| Mean hemoglobin,pg | 30.40 (29.20‒31.40) | 30.40(29.25‒31.40) | 30.20(28.90‒31.40) | 0.397 |
| ＜27 | 47 (8.36%) | 9(9.99%) | 38(8.41%) | 0.331 |
| 27‒31 | 277 (51.01%) | 44(48.35%) | 233(51.55%) |  |
| ＞31 | 162 (29.83%) | 28(30.77%) | 134(29.65%) |  |
| Mean hemoglobin concentration,g/L | 335.00 (326.01‒343.00) | 334.20(326.40‒342.67) | 330.00(320.12‒340.04) | 0.686 |
| ＜316 | 46 (8.66%) | 8(8.79%) | 38(8.41%) | 0.523 |
| 316‒354 | 419 (77.16%) | 72(79.12%) | 347(76.77%) |  |
| ＞354 | 22 (4.05%) | 2(2.20%) | 20(4.42%) |  |
| Red blood cell distribution width SD,fL | 41.00 (38.30‒44.00) | 41.88 (38.15‒44.43) | 40.10 (38.50‒41.75) | 0.037 |
| ＜35 | 12 (2.21%) | 3(3.30%) | 9(1.99%) | 0.267 |
| 35‒56 | 387 (71.27%) | 62(68.13%) | 325(71.90%) |  |
| ＞56 | 10 (1.84%) | 6(6.60%) | 10(2.21%) |  |
| Red blood cell distribution width CV, % | 12.50 (12.00‒13.30) | 12.60(12.00‒13.38) | 12.30(12.00‒13.20) | 0.717 |
| ＜11.5 | 23 (4.24%) | 3(3.30%) | 20(4.42%) | 0.569 |
| 11.5‒14.5 | 393 (72.38%) | 67(73.63%) | 326(72.12%) |  |
| ＞14.5 | 46 (8.47%) | 5(5.50%) | 41(9.07%) |  |
| Platelet count,10^9/L | 201.00 (152.00‒259.00) | 201.50(149.50‒258.00) | 200.00(150.44‒260.01) | 0.956 |
| <100 | 30 (5.52%) | 3(3.30%) | 27(5.97%) | 0.517 |
| 100‒300 | 384 (70.72%) | 65(71.43%) | 319(70.58%) |  |
| ＞300 | 67 (12.33%) | 13(14.29%) | 54(11.95%) |  |
| Platelet hematocrit, % | 0.21 (0.17‒0.26) | 0.21(0.17‒0.28) | 0.20(0.17‒0.25) | 0.686 |
| ＜0.10 | 11 (2.03%) | 3(3.30%) | 8(1.77%) | 0.665 |
| 0.10‒0.35 | 329 (60.59%) | 56(61.54%) | 273(60.40%) |  |
| ＞0.35 | 21 (3.87%) | 4(4.40%) | 17(3.76%) |  |
| Mean platelet volume, fl | 10.10 (9.33‒11.10) | 10.10(9.30‒11.20) | 10.20(9.40‒10.60) | 0.483 |
| ＜7 | 9 (1.66%) | 1(1.10%) | 8(1.77%) | 0.907 |
| 7‒13 | 415 (76.43%) | 69(75.82%) | 346(76.55%) |  |
| >13 | 30 (5.52%) | 5(5.50%) | 25(5.53%) |  |
| Platelet distribution width | 15.70 (12.00‒16.40) | 15.70(11.95‒16.40) | 15.80(12.10‒16.30) | 0.472 |
| <15 | 185 (34.07%) | 31(34.07%) | 154(34.07%) | 0.751 |
| 15~17 | 189 (34.81%) | 32(35.16%) | 157(34.73%) |  |
| >17 | 58 (10.68%) | 8(8.80%) | 50(11.06%) |  |
| Large platelet ratio, % | 27.60 (22.60‒34.60) | 27.50(22.55‒35.25) | 28.00(22.45‒31.95) | 0.589 |
| <10 | 1 (0.18%) | 0(0%) | 1(0.22%) | 0.909 |
| 10‒50 | 287 (52.85%) | 46(50.55%) | 241(53.32%) |  |
| >50 | 19 (3.50%) | 3(3.30%) | 16(3.54%) |  |
| Naive granulocyte percentage, % | 0.10 (0.03‒0.38) | 0.10(0.03‒0.48) | 0.15(0.023‒0.50) | 0.416 |
| ＜4 | 39 (7.18%) | 0(0%) | 31(6.86%) | 0.339 |
| ＞4 | 1 (0.18%) | 44(48.35%) | 1(0.22%) |  |
| Activated partial thromboplastin time, s | 31.00 (26.35‒34.10) | 30.60(26.60‒35.10) | 31.40(30.06‒35.80) | 0.687 |
| ＜31 | 131 (24.13%) | 21(23.08%) | 110(24.34%) | 0.785 |
| 31~43 | 115 (21.18%) | 22(24.18%) | 93(20.58%) |  |
| ＞43 | 20 (3.72%) | 4(4.40%) | 16(3.54%) |  |
| Thrombin time, s | 16.40 (14.95‒17.90) | 16.30(14.9‒17.85) | 16.60(15.05‒19.18) | 0.149 |
| >16 | 126 (23.20%) | 17(18.68%) | 109(24.12%) | 0.205 |
| 16‒18 | 92 (16.94%) | 16(17.58%) | 76(16.81%) |  |
| <18 | 63 (11.60%) | 15(16.48%) | 48(10.62%) |  |
| Fibrinogen, g/L | 3.19 (2.48‒3.97) | 3.16(2.44‒3.97) | 3.31(2.68‒4.03) | 0.461 |
| ＜1.7 | 10 (1.84%) | 3(3.30%) | 7(1.55%) | 0.842 |
| 1.7~4 | 201 (37.02%) | 32(35.16%) | 169(37.39%) |  |
| ＞4 | 69 (12.70%) | 12(13.19%) | 57(12.61%) |  |
| Prothrombin time, s | 12.20 (11.50‒13.20) | 12.30(11. 44‒13.23) | 12.05(11.50‒13.25) | 0.21 |
| ＜11 | 34 (6.26%) | 7(7.69%) | 27(5.97%) | 0.845 |
| 11‒13 | 169 (31.12%) | 28(30.77%) | 141(31.19%) |  |
| ＞13 | 78 (14.36%) | 13(14.29%) | 65(14.38%) |  |
| D-dimer,mg/L | 0.38(0.22‒0.77) | 0.38(0.21‒0.80) | 0.39(0.26‒0.66) | 0.368 |
| ＜0.5 | 137(25.23%) | 23(25.27%) | 111(24.56%) | 0.332 |
| ≥0.5 | 75(13.81%) | 17(18.68%) | 58(12.83%) |  |
| Body temperature during blood collection | 36.60(36.30‒36.80) | 36.70(36.23‒37.22) | 36.55(36.30‒36.80) | 0.401 |
| Blood oxygen concentration | 29.00(21.00‒39.00) | 30.00(23.25‒33.00) | 29.05(21.44‒39.21) | 0.198 |
| PH | 7.41(7.38‒7.44) | 7.43(7.40‒7.46) | 7.41(7.37‒7.44) | 0.756 |
| ＜7.35 | 1(0.19%) | 0(0%) | 1(0.2%) |  |
| 7.35‒7.45 | 64(11.9%) | 3(6.52%) | 61(12.4%) |  |
| ＞7.45 | 16(2.97%) | 1(2.17%) | 15(3.05%) |  |
| PH (T correction) | 7.40(7.38‒7.43) | 7.44(7.37‒7.49) | 7.40(7.38‒7.42) | 0.548 |
| CO2 partial pressure | 40.00(35.00‒43.00) | 37.50(32.50‒41.13) | 40.30(35.40‒43.00) | 0.176 |
| ＜35 | 20(3.72%) | 1(2.17%) | 19(3.86%) |  |
| 35‒45 | 59(10.97%) | 4(8.7%) | 55(11.18%) |  |
| ＞45 | 7(1.3%) | 0(0%) | 7(1.42%) |  |
| Oxygen saturation, % | 97.80(96.00‒99.00) | 96.30(94.00-98.45) | 98.00(96.00‒99.00) | 0.246 |
| Carbon dioxide partial pressure (T correction), mmHg | 41.00(37.60‒44.10) | 40.60(34.38‒44.43) | 41.00(37.85‒44.15) | 0.708 |
| ＜75 | 43(7.99%) | 2(4.35%) | 41(8.33%) |  |
| **Imaging features** |  |  |  |  |
| One side ground-glass opacity | 98 (18.05%) | 18 (19.78%) | 80 (17.70%) | 0.021 |
| Both sides ground-glass opacity | 345 (63.54%) | 57 (62.64%) | 288 (63.72%) | 0.039 |
| Consolidation | 12 (2.21%) | 0 (0%) | 12 (2.65%) | <0.001 |
| Others | 40 (7.37%) | 5 (5.49%) | 35 (7.74%) | 0.053 |
| Normal | 48 (8.84%) | 11 (12.09%) | 37 (8.19%) | 0.001 |
| Data are median (IQR), n (%), or n/N (%). p values were calculated by Mann-Whitney U test, χ² test, or Fisher’s exact test, as appropriate. | | | | |
